# Machine learning methods for predicting progression from mild cognitive impairment to Alzheimer’s disease dementia: a systematic review

**DOI:** 10.1186/s13195-021-00900-w

**Published:** 2021-09-28

**Authors:** Sergio Grueso, Raquel Viejo-Sobera

**Affiliations:** Cognitive NeuroLab, Faculty of Health Sciences, Universitat Oberta de Catalunya (UOC), Rambla del Poblenou 156, 08018 Barcelona, Spain

**Keywords:** Alzheimer’s disease, Conversion, Machine learning, Magnetic resonance, Mild cognitive impairment, PRISMA, Positron emission tomography, Prediction

## Abstract

**Background:**

An increase in lifespan in our society is a double-edged sword that entails a growing number of patients with neurocognitive disorders, Alzheimer’s disease being the most prevalent. Advances in medical imaging and computational power enable new methods for the early detection of neurocognitive disorders with the goal of preventing or reducing cognitive decline. Computer-aided image analysis and early detection of changes in cognition is a promising approach for patients with mild cognitive impairment, sometimes a prodromal stage of Alzheimer’s disease dementia.

**Methods:**

We conducted a systematic review following PRISMA guidelines of studies where machine learning was applied to neuroimaging data in order to predict whether patients with mild cognitive impairment might develop Alzheimer’s disease dementia or remain stable. After removing duplicates, we screened 452 studies and selected 116 for qualitative analysis.

**Results:**

Most studies used magnetic resonance image (MRI) and positron emission tomography (PET) data but also magnetoencephalography. The datasets were mainly extracted from the Alzheimer’s disease neuroimaging initiative (ADNI) database with some exceptions. Regarding the algorithms used, the most common was support vector machine with a mean accuracy of 75.4%, but convolutional neural networks achieved a higher mean accuracy of 78.5%. Studies combining MRI and PET achieved overall better classification accuracy than studies that only used one neuroimaging technique. In general, the more complex models such as those based on deep learning, combined with multimodal and multidimensional data (neuroimaging, clinical, cognitive, genetic, and behavioral) achieved the best performance.

**Conclusions:**

Although the performance of the different methods still has room for improvement, the results are promising and this methodology has a great potential as a support tool for clinicians and healthcare professionals.

## Background

The increase in lifespan experienced in Western societies has largely been driven by medical and technological advances [1]; however, this improvement has resulted in an increasing number of people diagnosed with neurocognitive disorders. In 2010, dementia was associated with $604 billion of healthcare expenses in the USA [2]. Every year, ten million new cases of dementia are registered, and by 2050, it is estimated that 135 million people will have some degree of dementia [3]. Age is the main risk factor for dementia; the prevalence is 1–2% at the age of 65 but reaches 30% at the age of 85. From all neurodegenerative disorders, about 60–90% are characterized as Alzheimer’s disease (AD) dementia subtype (depending on the diagnostic criteria used) [4], for which there is yet no cure.

Patients are typically diagnosed with AD when the symptoms of a cognitive decline have already manifested, i.e., when dementia has appeared. In such cases, the diagnosis is determined too late, failing to implement preventive protocols to reduce cognitive decline. Pharmacological and non-pharmacological treatments have proven to be effective in reducing cognitive and behavioral symptoms in the early stages of the disease [5]. In light of these treatments, recent studies have focused on detecting patients with cognitive impairment that have not reached dementia in order to delay or prevent its development. The last edition of the Diagnostic and Statistical Manual of Mental Disorders (DSM-5) includes a specific category for this type of patients called a mild neurocognitive disorder, analogous to the mild cognitive impairment (MCI) whose main characteristic is having minor memory impairment [4] (throughout this review, MCI will be used instead of mild neurocognitive disorder as it is more frequent in the scientific literature). MCI can, in some cases, be a prodromal stage of dementia, especially for AD [6]. It is worth mentioning at this point that AD should be considered as a continuum, where patients with MCI that will eventually progress to AD dementia already have AD, but the cognitive symptoms have not yet fully manifested. For this reason, it is important to differentiate between those MCI patients that will progress to AD dementia and those who will remain stable.

In late stages, when dementia symptoms have already appeared, AD is easier to confirm with neuroimaging techniques and cerebrospinal fluid evaluations for the presence of neurofibrillary clews, beta-amyloid and tau proteins [7], and temporal cortex atrophy [4]. Nevertheless, in the early stages, although biomarkers may be present in magnetic resonance image (MRI) and/or positron emission tomography (PET) results, the detection of MCI to AD dementia progression remains challenging in the clinical practice [8, 9]. To overcome this challenge, the scientific community now has access to thousands of neuroimaging longitudinal datasets from healthy, MCI, and AD subjects along with other variables (i.e., demographic, genetic, and cognitive measurements, etc.) stored in public databases such as the Alzheimer disease neuroimaging initiative (ADNI) (http://adni.loni.usc.edu). These datasets can be compared and analyzed to perform classification and automatic detection of AD and MCI progression [10, 11] using newly developed computer-aided techniques like machine learning (ML) algorithms. Then, these new tools could be transferred to the clinic to assist in the early diagnosis and prognosis.

The ML paradigm consists of training an algorithm with a dataset; in this case, neuroimaging results together with other clinical variables, to extract common factors that help classify subjects according to a variable of interest. In the case of an early diagnosis of AD and distinction from a stable MCI condition, for example, the algorithm learns to classify the data according to the specific diagnosis and extracts which factors have been the most relevant for the differentiation between the groups. Subsequently, the trained algorithm can be used to classify a specific individual for which we do not know the diagnosis and thus manage to assist in the therapeutic approach [12–14]. This technique can be applied to any disease that occurs with morphological changes or with characteristic neural patterns. See Arbabshirani, Plis, Sui, & Calhoun [15] for a review of the same objective and methodology but applied to autism, attention deficit disorder, and schizophrenia.

Recent work has demonstrated that ML algorithms are able to classify images from AD, MCI, and healthy participants with very high accuracy levels [16, 17]. Although such classification has provided valuable information about AD biomarkers, for this technology to have a more substantial clinical impact by empowering a clinician to administer a customized treatment protocol, it is necessary to determine and predict whether a MCI patient will progress to AD dementia or remain stable. The goal of this systematic review is to analyze the existing classification methods based in ML algorithms applied to neuroimaging data in combination with other variables for predicting MCI to AD dementia progression.

## Methods

To perform this systematic review, we followed the Preferred Reporting Items for Systematic Reviews and Meta-Analysis (PRISMA) guidelines [18, 19]. A systematic search was done to find studies that included ML methods to predict MCI to AD dementia progression using neuroimaging techniques. Progression to AD dementia from MCI is established when, during a follow-up period (3 years for ADNI and 1 year for AddNeuroMed databases), a patient that was initially classified as MCI, is diagnosed with Alzheimer (that is a “progressive MCI” or pMCI) based on clinical criteria (MMSE and CDR scales, and NINCDS/ADRDA criteria for probable AD dementia [20, 21]). Patients are considered “stable MCI” (sMCI), when they were diagnosed as MCI at baseline and the diagnosis remained as MCI during the follow-up.

Only articles written in English and published between January 2010 and May 2021 (included) were selected. Articles published before 2010 were not included because the technological (e.g., computational power, graphical processing units) and methodological (e.g., ML and deep learning algorithm development) gap between those studies and the current standards make them hardly comparable. In fact, even the comparison between articles published in the early vs. the late 2010s presents methodological gaps. These differences are not only due to better methods but mainly to technological advances that were not possible before and early 2010s, and the growth of the ADNI database.

We performed an advanced search concatenating terms with Boolean operators in PubMed, PsycINFO, and Web of Science databases as follows: (“computational neuroscience” OR “artificial intelligence” OR “machine learning” OR “deep learning” OR “neural network*”) AND (“neuroimag*”) AND (“Alzheimer*” OR “AD dementia”) AND (“mild cognitive impairment” OR “MCI”) AND (“conversion” OR “predict*” OR “follow-up”).

After removing duplicates, the eligibility criteria were applied by two independent reviewers (SG and RVS) to select only the articles that included (1) prediction of MCI to AD dementia conversion, (2) use of neuroimaging data, (3) classification methods based on ML algorithms, and (4) accuracy results.

Once the selection of studies was concluded, the following data was extracted for each study: (1) first author and year of publication, (2) groups, (3) sample size and mean age, (4) database, (5) neuroimaging technique used and variables selected, (6) classification method, (7) validation method, (8) accuracy achieved, and (9) area under the ROC curve.

We also analyzed the risk of bias of the selected studies. The aspects considered in the analysis of bias were based on the Cochrane guidelines for systematic reviews [22], but the exact criteria were adapted by taking into account the particular methodology and goals of the studies, focused on creating and validating a classification model in large datasets. The criteria used are detailed in Table 1.

**Table 1:** Risk of bias analysis criteria

Risk of bias	Score	Criteria
Database	Low (0)	Use of validated and widely used dataset for the study of biomarkers of Alzheimer’s disease (AD) including several years of follow-up with information of stable and progressive MCI patients
	Medium (1)	Use of similar database with less widespread usage
	High (2)	The participants were selected by the authors and no validated database was used
Validation of the classification method	Low (0)	The study validates the classification method with a test sample and/or an independent sample
	Medium (1)	It uses a different validation method
	High (2)	There is no validation of the classification method
Mathematical development of the algorithms	Low (0)	Explanation of their theoretical basis or architecture for neural networks
	Medium (1)	The authors refer to literature but do not develop their mathematical notation or architecture
	High (2)	No information about the model

We also performed an interpretability analysis based on the framework proposed by Kohoutová et al. [23]. These authors have developed three levels of assessment for the interpretability of ML models in neuroimaging based on the model itself, the feature selection and characteristics, and biological factors; also, each level has several sublevels. *Model-level* assessment consists of evaluating the model as a whole and testing it in different contexts and conditions. The sublevels include sensitivity and specificity, generalizability, behavioral analysis, representational analysis, and analysis of confounds. *Feature-level* assessment consists of evaluating the significance of individual features used in prediction, including stability, feature importance, and visualization. Finally, the *biology-level* assessment is a validation of the model based on its neurobiological plausibility and it has two sublevels: literature (relationship of the model with previous literature) and invasive studies (the possibility of using more invasive methods).

We assessed whether the studies included in the review complied with each of the sublevels, but we did not include behavioral analysis, representational analysis, and invasive studies sub-levels. *Behavior analysis* sub-level was not considered because the only “behavior” of the model is to classify subjects, and the behavior is measured as accuracy, which is included in the sensitivity and specificity sublevels. *Representational analysis* compares the model with other models, other brain regions, or other experimental settings; in our review, the main goal of almost all studies was to find neural patterns that predict AD dementia, and therefore, it is common to use the whole brain as a feature. Also, there is only one experimental setting aimed to find maximum classification accuracy so it cannot be compared to similar experiments in the same study, only with similar literature (which represents another sub-level). Finally, the *invasive studies* sub-level is not applicable because the long-term objective of these investigations is to find a non-invasive method of predicting AD dementia as soon as possible.

## Results

**Fig. 1:**
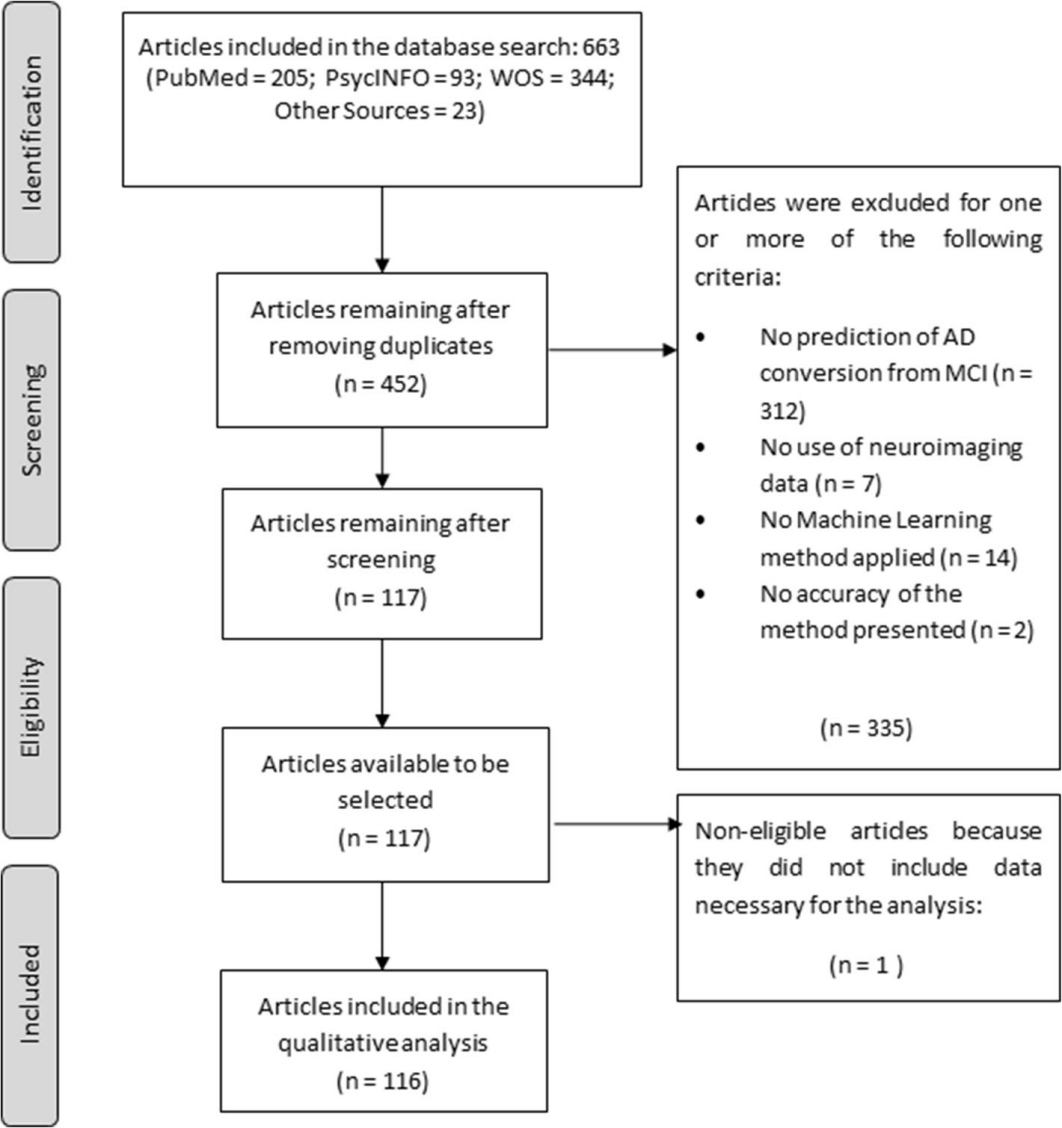
PRISMA workflow for study selection adapted from Moher et al. [18]. WOS Web Of Science, AD Alzheimer’s disease, MCI mild cognitive impairment

As shown in Fig. 1, the workflow followed for the article selection included the four phases (identification, screening, eligibility, and inclusion) proposed by the PRISMA guidelines [18, 19]. The 452 articles remaining after eliminating duplicates were screened, and after applying the exclusion criteria, 117 articles were selected for the review.

**Fig. 2:**
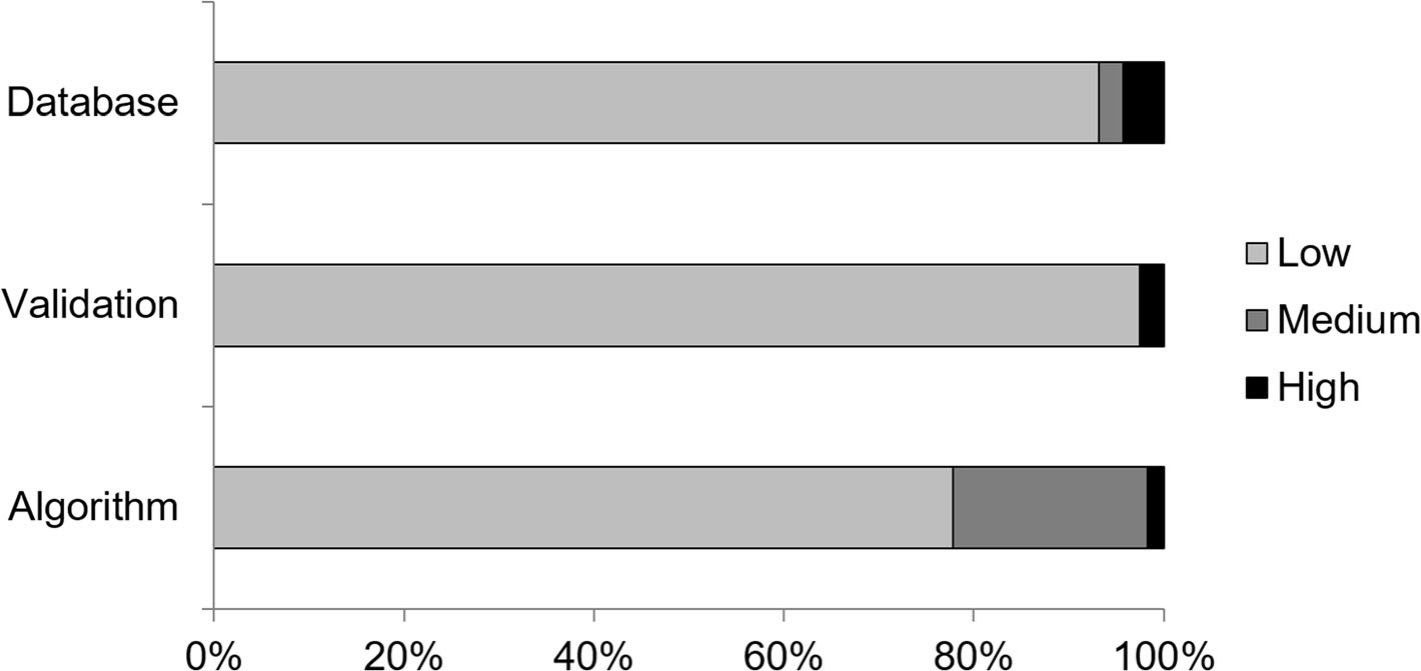
Risk of bias analysis. Percentage of studies presenting low, medium or high risk of bias in each of the categories analyzed

The risk of bias analysis is shown in Fig. 2 and Table 2. The overall risk of bias of all the studies was considered low. From the 117 articles selected at the eligibility stage, only one study [65] was not included in the qualitative analysis because of the high risk of bias. The sample size in this study was seven subjects, and it did not include any validation method. Therefore, the final number of studies included in the qualitative analysis was 116.

**Table 2:** Risk of bias analysis for individual studies

Author (year)	Algorithm	Validation	Database	Total bias
Plant et al. (2010) [24]	0	0	2	2
Chincarini et al. (2011) [25]	0	0	0	0
Costafreda et al. (2011) [26]	1	0	1	2
Filipovych et al. (2011) [27]	0	0	0	0
Hinrichs et al. (2011) [8]	0	0	0	0
Westman et al. (2011) [28]	0	0	1	1
Wolz et al. (2011) [29]	1	0	0	0
Zhang et al. (2011) [30]	0	0	0	0
Batmanghelich et al. (2012) [31]	0	0	0	0
Cheng et al. (2012) [32]	0	0	0	0
Cho et al. (2012) [33]	0	0	0	0
Gray et al. (2012) [34]	0	0	0	0
Li et al. (2012) [35]	1	0	0	1
Toussaint et al. (2012) [36]	1	0	0	1
Wee et al. (2012) [37]	0	0	0	0
Ye et al. (2012) [38]	1	0	0	1
Zhang et al. (2012) [9]	0	0	0	0
Adaszewski et al. (2013) [39]	1	0	0	1
Aguilar et al. (2013) [40]	1	0	1	2
Babu et al. (2013) [41]	0	0	0	0
Casanova et al. (2013) [42]	0	0	0	0
Cheng et al. (2013) [43]	0	0	0	0
Liu, M. et al. (2013) [44]	0	0	0	0
Liu, X. et al. (2013) [45]	0	0	0	0
Wee et al. (2013) [46]	0	0	0	0
Young et al. (2013) [47]	1	0	0	1
Apostolova et al. (2014) [48]	1	0	0	1
Guerrero et al. (2014) [49]	0	0	0	0
Lebedev et al. (2014) [50]	0	0	0	0
Liu, M. et al. (2014) [51]	0	0	0	0
Liu, F. et al. (2014) [52]	0	0	0	0
Min et al. (2014) [53]	0	0	0	0
Suk et al. (2014) [54]	0	0	0	0
Tong et al. (2014) [55]	0	0	0	0
Cabral et al. (2015) [56]	0	0	0	0
Cheng et al. (2015) [57]	0	0	0	0
Cheng et al. (2015) [58]	0	0	0	0
Moradi et al. (2015) [59]	0	0	0	0
Raamana et al. (2015) [60]	0	0	0	0
Ritter et al. (2015) [61]	1	0	0	1
Salvatore et al. (2015) [62]	0	0	0	0
Xu et al. (2015) [63]	0	0	0	0
Ardekani et al. (2016) [64]	1	1	0	2
Cappeci et al. (2016) [65]	0	2	2	4
Collij et al. (2016) [66]	0	0	2	2
Li et al. (2016) [67]	2	0	0	2
Liu et al. (2016) [68]	0	0	0	0
López et al. (2016) [69]	0	0	0	0
Suk et al. (2016) [70]	0	0	0	0
Thung et al. (2016) [71]	0	0	0	0
Vasta et al. (2016) [72]	0	0	0	0
Zhang et al. (2016) [73]	0	0	0	0
Zhang et al. (2016) [74]	0	0	0	0
Ҫitak-Er et al. (2017) [75]	0	0	0	0
Hojjati et al. (2017) [76]	0	0	0	0
Long et al. (2017) [77]	0	0	0	0
Mathotaarachchi et al. (2017) [78]	0	0	0	0
Suk et al. (2017) [79]	0	0	0	0
Tong et al. (2017) [80]	0	0	0	0
Choi et al. (2018) [81]	0	0	0	0
Donnelly-Kehoe et al. (2018) [82]	1	0	0	1
Gao et al. (2018) [83]	1	0	0	1
Gómez-Sancho et al. (2018) [84]	0	0	0	0
Hojjati et al. (2018) [85]	1	0	0	1
Khanna et al. (2018) [86]	0	0	0	0
Lin et al. (2018) [87]	0	0	0	0
Liu et al. (2018) [88]	0	0	0	0
Liu et al. (2018) [89]	0	0	0	0
Lu et al. (2018) [90]	0	0	0	0
Minhas et al. (2018) [91]	0	0	0	0
Popuri et al. (2018) [92]	2	0	0	2
Sorensen et al. (2018) [93]	0	0	0	0
Sun et al. (2018) [94]	0	0	0	0
Wu et al. (2018) [95]	0	0	0	0
Yan et al. (2018) [96]	0	0	0	0
Basaia et al. (2019) [97]	0	0	0	0
Cheng et al. (2019) [98]	0	0	0	0
Collazos-Huertas et al. (2019) [99]	1	0	0	1
Elahifasaee et al. (2019) [100]	0	0	0	0
Ezzati et al. (2019) [101]	1	0	0	1
Gupta et al. (2019) [102]	0	0	0	0
Lee et al. (2019) [103]	0	0	0	0
Lee et al. (2019) [104]	0	0	0	0
Lei et al. (2019) [105]	0	0	0	0
Li et al. (2019) [106]	0	0	0	0
Li et al. (2019) [107]	0	0	0	0
Oh et al. (2019) [108]	0	0	0	0
Pan et al. (2019) [109]	0	0	0	0
Pusil et al. (2019) [110]	0	0	2	2
Spasov et al. (2019) [111]	0	0	0	0
Wang et al. (2019) [112]	1	0	0	1
Wee et al. (2019) [113]	0	0	0	0
Xu et al. (2019) [114]	0	0	0	0
Zhou et al. (2019) [115]	0	0	0	0
Zhu et al. (2019) [116]	0	0	0	0
Abrol et al. (2020) [117]	0	0	0	0
Gao et al. (2020) [118]	0	0	0	0
Giorgio et al. (2020) [119]	1	0	0	1
Khatri et al. (2020) [120]	0	0	0	0
Lin et al. (2020) [121]	0	0	0	0
Lin et al. (2020) [122]	0	0	0	0
Pan et al. (2020) [123]	0	0	0	0
Ramon-Julvez et al. (2020) [124]	1	0	0	1
Xiao et al. (2020) [125]	0	0	0	0
Xu et al. (2020) [126]	0	0	2	2
Yang et al. (2020) [127]	1	0	0	1
Yee et al. (2020) [128]	0	0	0	0
Zhou et al. (2020) [129]	0	0	0	0
Bae et al. (2021) [130]	0	0	0	0
Mofrad et al. (2021) [131]	1	0	0	1
Mofrad et al. (2021) [132]	1	0	0	1
Pan et al. (2021) [133]	0	0	0	0
Shen et al. (2021) [134]	0	0	0	0
Syaifullah et al. (2021) [135]	1	0	0	1
Wen et al. (2021) [136]	1	0	0	1
Zhang et al. (2021) [137]	0	0	0	0
Zhu et al. (2021) [138]	0	0	0	0
Total	28/234	3/234	13/234	43/702

Note. This table shows the results of the bias analysis performed based on Higgins et al. [22] with the punctuations specified in Table 1

The studies selected for the qualitative analysis are presented in Table 3 following the structure explained in the data extraction section (study, cohort, sample [mean age], database, features and neuroimaging technique, classification method, validation method, results [% accuracy], and AUC ROC).

**Table 3:** Studies selected following PRISMA guidelines presented in chronological order

Author (year)	Groups	Sample size (mean age)	Database	Neuroimaging technique and features	Classification method	Validation method	Results (% accuracy)	AUC ROC
Plant et al. (2010) [24]	HS AD MCI	18 (64.8) 32 (68.8) 24 (69.7)	Sample collected for the study	MRI: whole-brain volume measures	SVM Bayes VFI	Train/test method: AD + HS as train set and MCI as test set.	pMCI vs sMCI: SVM: 50 Bayes: 58.3 VFI: 75	NA
Chincarini et al. (2011) [25]	HS AD sMCI pMCI	189 (76.6) 144 (75.5) 166 (75.7) 136 (75.1)	ADNI-1	MRI: GM volumes	SVM	20-fold Cross Validation	NA	0.74
Costafreda et al. (2011) [26]	HS AD MCI	88 (73.6) 71 (74.9) 103 (74.1)	AddNeuroMed	MRI: 3D hippocampal morphometric measures	SVM with RBF kernel	4-fold Cross Validation	pMCI vs sMCI: 80	NA
Filipovych et al. (2011) [27]	HS AD sMCI pMCI	63 (75.2) 54 (77.4) 174 (74.5) 68 (76.2)	ADNI-1	MRI: whole-brain GM density	Semi-supervised SVM	*Leave-one-out* Cross Validation	pMCI: 79.4 sMCI: 51.7	0.69
Hinrichs et al. (2011) [8]	HS AD MCI	66 (76.2) 48 (76.6) 119 (75.1)	ADNI-1	MRI and PET: scan data, APOE4 genotype, CSF assays, and cognitive tests	MK-SVM	Train/test method: AD + HS as train set and MCI as test set	pMCI vs sMCI: NA	0.79
Westman et al. (2011) [28]	HS AD MCI	112 (73) 117 (76) 122 (75)	AddNeuroMed	MRI: whole-brain volume, age, and education	OPLS	Train/test method: sample of 51 subjects	pMCI vs sMCI: 73	NA
Wolz et al. (2011) [29]	HS AD sMCI pMCI	231 (76) 198 (75.7) 238 (74.8) 167 (74.6)	ADNI-1	MRI: hippocampal volume, cortical thickness, tensor-based morphometry, and manifold-based learning	SVM LDA	Train/test method: 95/5 partition	pMCI vs sMCI: SVM: 60 LDA: 68	NA
Zhang et al. (2011) [30]	HS AD sMCI pMCI	52 (75.3) 51 (75.2) 56 (75.3) 43 (75.3)	ADNI-1	MRI and PET: volume, intensity, and CSF (Aβ_42,_ t-tau y p-tau) measures	SVM	10-fold cross-validation	pMCI: 91.5 sMCI: 73.4	NA
Batmanghelich et al. (2012) [31]	sMCI pMCI	139 (NA) 99 (NA)	ADNI-1	MRI: WM, GM, and CSF	Logistic model trees + Laplacian SVM	5-fold cross-validation	pMCI vs sMCI: 61.5	NA
Cheng et al. (2012) [32]	HS AD sMCI pMCI	52 (75.3) 51 (75.2) 56 (75.3) 43 (75.3)	ADNI-1	MRI and PET: GM and WM volume, intensity, and CSF (Aβ_42,_ t-tau y p-tau) measures	Domain Transfer SVM	Train/test method: AD + HS as train set and MCI as test set with 10-fold cross-validation	pMCI vs sMCI: 69.4	0.74
Cho et al. (2012) [33]	HS AD sMCI pMCI	160 (76.2) 128 (76) 131 (74.1) 72 (74.8)	ADNI-1	MRI: cortical thickness	LDA	Train/test method: 50/50 partition	pMCI vs sMCI: 70	NA
Gray et al. (2012) [34]	HS AD sMCI pMCI	54 (NA) 50 (NA) 64 (NA) 53 (NA)	ADNI-1	PET: signal intensity and relative change over 12 months	SVM with RBF kernel	Train/test method: 75/25 partition with 1000 iterations	pMCI vs sMCI: 63.1	0.66
Li et al. (2012) [35]	HS AD sMCI pMCI	40 (73.7) 37 (74.8) 36 (75.3) 39 (75.6)	ADNI-1	MRI: static and dynamic cortical thickness and clustering coefficient	SVM	*Leave-one-out* cross-validation	pMCI vs sMCI: 81.7	NA
Toussaint et al. (2012) [36]	HS AD sMCI pMCI	80 (76.4) 80 (76) 40 (76.4) 40 (76.4)	ADNI-1	PET: glucose metabolic signal and clinical measures	Two-sample t-test + spatial ICA + SVM with RBF kernel	*Leave-one-out* cross-validation	pMCI vs sMCI: 80	NA
Wee et al. (2012) [37]	HS MCI	17 (72.1) 10 (74.2)	ADNI-1	MRI and PET: WM structural connectivity and GM functional connectivity	Mk-SVM	*Leave-one-out* cross-validation	pMCI vs sMCI: 96.3	0.95
Ye et al. (2012) [38]	sMCI pMCI	177 (NA) 142 (NA)	ADNI-1	MRI: GM and WM volumes, cortical thickness, demographic, genetic, and cognitive measures	SVM	*Leave-one-out* Cross Validation	pMCI vs sMCI: NA	0.85
Zhang et al. (2012) [9]	HS AD sMCI pMCI	50 (75.3) 45 (75,4) 48 (74.7) 43 (75.8)	ADNI-1	MRI and PET: volume, intensity, and CSF (Aβ_42,_ t-tau y p-tau) measures	M3TL	10-fold cross-validation	pMCI vs sMCI: 73.9	0.80
Adaszewski et al. (2013) [39]	HS AD sMCI pMCI	137 (NA) 108 (NA) 61 (74) 142 (74)	ADNI-1 ADNI-GO	MRI: whole-brain GM volume	SVM	Train/test method: AD + HS subset as train set and bootstrapping with 100 permutations	pMCI: 63.7 sMCI: NA	NA
Aguilar et al. (2013) [40]	HS AD sMCI pMCI	110 (73) 116 (74.4) 98 (74.7) 21 (72.9)	AddNeuroMed	MRI: volume and cortical thickness	OPLS SVM Decision Trees ANN	10-fold cross-validation	pMCI vs sMCI: OPLS: 74.7 SVM: 70.9 Decision Trees: 67.4 ANN: 70.1	0.83 0.81 0.80 0.75
Babu et al. (2013) [41]	HS sMCI pMCI	232 (76) 236 (74.9) 167 (74.6)	ADNI-1	MRI: GM volumes	PBL-McqRBFN	Train/test method: 95/5 partition	pMCI vs sMCI: 79	NA
Casanova et al. (2013) [42]	HS AD sMCI pMCI	188 (75.9) 171 (75.5) 182 (75.2) 153 (75)	ADNI-1	MRI: GM volumes	RLR	10-fold cross-validation	pMCI vs sMCI: 61.5	NA
Cheng et al. (2013) [43]	HS AD sMCI pMCI	52 (NA) 51 (NA) 56 (NA) 43 (NA)	ADNI-1	MRI and PET: volume, intensity, APOE4 genotype, and CSF (Aβ_42,_ t-tau y p-tau) measures	SM2TLC	10-fold cross-validation	pMCI vs sMCI: 77.8	0.81
Liu, M. et al. (2013) [44]	sMCI pMCI	185 (74.9) 164 (74.9)	ADNI-1	MRI: GM volumes	MTSRC	*Leave-one-out* cross-validation	pMCI vs sMCI: 74.1	0.75
Liu, X. et al. (2013) [45]	HS AD sMCI pMCI	138 (76) 86 (75) 93 (75) 97 (75)	ADNI-1	MRI: volume and cortical thickness	SVM EN LDA	*Leave-one-out* cross-validation	pMCI vs sMCI: SVM: 66 EN: 68 LDA: 68	0.53 NA 0.68
Wee et al. (2013) [46]	HS AD sMCI pMCI	200 (75.8) 198 (75.7) 111 (75.3) 89 (74.8)	ADNI-1	MRI: cortical thickness and correlation of cortical thickness between pairs of ROIs	Mk-SVM	10-fold cross-validation	pMCI vs sMCI: 75	0.84
Young et al. (2013) [47]	HS AD sMCI pMCI	73 (75.9) 63 (75.2) 96 (75.6) 47 (74.5)	ADNI-1	MRI and PET: volume, intensity, APOE4 genotype, and CSF (Aβ_42,_ t-tau y p-tau) measures	Gaussian Process	*Leave-one-out* cross-validation	pMCI vs sMCI: 74.1	0.80
Apostolova et al. (2014) [48]	HS AD MCI	111 (NA) 95 (NA) 182 (NA)	ADNI-1	MRI: hippocampal volumes and demographic, APOE genotype, and CSF measures	SVM	*Leave-one-out* cross-validation	pMCI vs sMCI: 68	0.68
Guerrero et al. (2014) [49]	HS AD sMCI pMCI	175 (76.3) 106 (75.4) 114 (75.1) 116 (74.7)	ADNI-1 ADNI-GO	MRI: 3D brain volumes	SVM	Train/test method: unknown partition	pMCI vs sMCI: 97.2	NA
Lebedev et al. (2014) [50]	HS AD MCI	225 (75.9) 185 (75.2) 165 (75.5)	ADNI-1	MRI: cortical thickness, demographic variables, and APOE4 genotype	RF	Independent test set	pMCI vs sMCI: 82.3	0.83
Liu, M. et al. (2014) [51]	HS AD sMCI pMCI	229 (76) 198 (75.7) 236 (74.9) 167 (74.9)	ADNI-1	MRI. whole-brain GM density	SVM	10-fold cross-validation	pMCI vs sMCI: 70.7	NA
Liu, F. et al. (2014) [52]	HS AD MCI	52 (75.3) 51 (75.2) 99 (75.3)	ADNI-1	MRI and PET: volume and intensity measures	Mk-SVM	10-fold cross-validation	pMCI vs sMCI: 67.8	0.70
Min et al. (2014) [53]	HS AD sMCI pMCI	128 (76.1) 97 (75.9) 117 (75.1) 117 (75.2)	ADNI-1	MRI: multi-atlas GM volume measures	SVM	10-fold cross-validation	pMCI vs sMCI: 72.4	0.67
Suk et al. (2014) [54]	HS AD MCI	101 (75.9) 93 (75.5) 204 (74.9)	ADNI-1	MRI and PET: volume and intensity measures	DBM	10-fold cross-validation	pMCI vs sMCI: 75.9	0.75
Tong et al. (2014) [55]	HS AD sMCI pMCI	231 (76) 198 (75.7) 238 (74.9) 167 (74.6)	ADNI-1	MRI: intensity patches	Multiple instance-graph	*Leave-one-out* cross-validation	pMCI vs sMCI: 70.4	NA
Cabral et al. (2015) [56]	sMCI pMCI	56 (NA) 44 (NA)	ADNI-1	PET: voxel intensities	Linear-SVM SVM-RBF Gaussian Naïve Bayes	10-fold cross-validation	pMCI vs sMCI: Linear-SVM: 71–89 SVM-RBF: 75–85 Gaussian Naïve Bayes: 73–81	NA
Cheng et al. (2015) [57]	HS AD sMCI pMCI	52 (NA) 51 (NA) 56 (NA) 53 (NA)	ADNI-1	MRI and PET: volume, intensity and CSF (Aβ_42,_ t-tau y p-tau) measures	M2TL	10-fold cross-validation	pMCI vs sMCI: 80.1	0.85
Cheng et al. (2015) [58]	HS AD sMCI pMCI	52 (NA) 51 (NA) 56 (NA) 43 (NA)	ADNI-1	MRI and PET: volume, intensity, APOE4 genotype, and CSF (Aβ_42,_ t-tau y p-tau) measures	Domain Transfer SVM	10-fold cross-validation	pMCI vs sMCI: 79.4	0.85
Moradi et al. (2015) [59]	HS AD sMCI pMCI	231 (NA) 200 (NA) 100 (NA) 164 (NA)	ADNI-1	MRI: GM volumes, age, and cognitive measures	RF	10-fold cross-validation	pMCI vs sMCI: 81	0.90
Raamana et al. (2015) [60]	HS AD sMCI pMCI	159 (NA) 136 (NA) 130 (NA) 56 (NA)	ADNI-1	MRI: cortical thickness	Variational Bayes probabilistic MKL	Train/test method: 95/5 partition	pMCI vs sMCI: 64	0.68
Ritter et al. (2015) [61]	sMCI pMCI	151 (74.1) 86 (74.6)	ADNI-1	MRI and PET: cortical thickness, intensity measurements, neuropsychological tests, clinical variables, and demographic data	SVM with RBF kernel Classification tree RF	30 iterations of 10-fold cross-validation	pMCI vs sMCI: SVM: 61-64 Classification Tree: 61-64 RF: 60-61	NA
Salvatore et al. (2015) [62]	HS AD sMCI pMCI	162 (76.3) 137 (76) 134 (74.5) 76 (74.8)	ADNI-1	MRI: GM and WM volumes	SVM	20-fold cross-validation	pMCI vs sMCI: 66	NA
Xu et al. (2015) [63]	HS AD MCI	117 (75.4) 113 (75.6) 110 (75.2)	ADNI-1	MRI and PET: volume and intensity measures	wmSRC	10-fold cross-validation	pMCI vs sMCI: 77.8	0.80
Ardekani et al. (2016) [64]	sMCI pMCI	78 (NA) 86 (NA)	ADNI-1	MRI: hippocampal volumetric integrity, APOE genotype, demographic data, and neuropsychological tests	RF	Out-of-bag method	pMCI vs sMCI: 82.3	0.83
Collij et al. (2016) [66]	HS AD MCI	100 (61.7) 100 (63.1) 60 (62.7)	Sample collected for the study	MRI: whole-brain arterial spin labeling perfusion maps	SVM	Train/test method: 50/50 partition	pMCI vs sMCI: 70.8	0.77
Li et al. (2016) [67]	HS AD sMCI pMCI	42 (65.6) 25 (69.4) 10 (66.5) 21 (68.6)	ADNI-1	MRI: GM whole-brain and seed-based functional connectivity	SVM	*Leave one out* cross-validation	pMCI vs SMCI: 80.6	NA
Liu et al. (2016) [68]	HS AD sMCI pMCI	97 (75.9) 128 (76.1) 117 (75.1) 117 (75.2)	ADNI-1	MRI: GM density maps	SVM	10-fold cross-validation	pMCI vs SMCI: 79.2	0.83
López et al. (2016) [69]	sMCI pMCI	21 (72.7) 12 (75.7)	Sample collected for the study	MRI and MEG: MEG power data on each ROI and hippocampal volumes, age, gender, cognitive reserve, neuropsychological testing score, and APOE genotype	HLR	Train/test method: 75/25 partition	pMCI vs sMCI: 100	0.97
Suk et al. (2016) [70]	HS AD sMCI pMCI	52 (75.3) 51 (75.2) 56 (75) 43 (75.7)	ADNI-1	MRI and PET: GM, CSF, and intensity measures	DW-S2MTL	10-fold cross validation	pMCI vs sMCI: 73	NA
Thung et al. (2016) [71]	sMCI pMCI	53 (75.7) 60 (75.2)	ADNI-1	MRI: whole-brain GM volume and changes in 4 years of follow-up	Mk-SVM	10-fold cross validation	pMCI vs sMCI: 78.2	0.84
Vasta et al. (2016) [72]	HS AD sMCI pMCI	47 (78.2) 55 (75.9) 89 (75.4) 32 (75.5)	ADNI-1	MRI: hippocampal volumes	SVM Naive Bayes Neural Network	Train/test method: AD + HS as train set and MCI as test set	pMCI vs sMCI: SVM: 66.1 Naïve Bayes: 65.3 Neural Network: 66.9	NA NA NA
Zhang et al. (2016) [73]	sMCI pMCI	62 (75.4) 71 (74.8)	ADNI-1	MRI: whole ventricular tensor-based morphometry	SVM	5-fold cross-validation	pMCI vs sMCI: 96.7	0.97
Zhang et al. (2016) [74]	AD HS MCI	194 (NA) 228 (NA) 388 (NA)	ADNI-1	MRI: multivariate hippocampal surface TBM and radial distance	AdaBoost	*Leave-one-out* cross-validation	pMCI vs sMCI: 77	0.75
Ҫitak-Er et al. (2017) [75]	sMCI pMCI	165 (70.9) 140 (73.1)	ADNI-1	MRI: GM volumes	Linear SVM Polynomial-SVM LR	10-fold cross-validation	pMCI vs sMCI: Linear-SVM: 73.1 Poly-SVM: 78.7 LR: 76.1	NA
Hojjati et al. (2017) [76]	sMCI pMCI	62 (73) 18 (73.6)	ADNI-1	MRI: connectivity matrix	SVM	9-fold cross-validation	pMCI vs sMCI: 91.4	0.95
Long et al. (2017) [77]	HS AD sMCI pMCI	135 (76.2) 65 (75.6) 132 (75.2) 95 (75.1)	ADNI-1	MRI: whole-brain GM and WM	SVM	10-fold cross-validation	pMCI vs sMCI: with GM: 85.9 with WM: 68.7	GM: 0.89 WM: 0.68
Mathotaarachchi et al. (2017) [78]	sMCI pMCI	230 (71.4) 43 (73.2)	ADNI-1	PET: intensity, demographic, and AOPE4 genotype measures	RUSRF	10-fold cross-validation	pMCI vs sMCI: 84	0.91
Suk et al. (2017) [79]	HS AD sMCI pMCI	226 (NA) 186 (NA) 226 (NA) 167 (NA)	ADNI-1	MRI: GM volume	CNN	10-fold cross-validation	pMCI vs sMCI: 74.8	0.75
Tong et al. (2017) [80]	HS AD sMCI pMCI	229 (75.9) 191 (75.3) 129 (74.6) 171 (74.5)	ADNI-1	MRI: global grading biomarker, age, and cognitive measures	SVM	10-fold cross-validation	pMCI vs sMCI: 81	0.87
Choi et al. (2018) [81]	HS AD sMCI pMCI	182 (73.4) 139 (74.3) 92 (70.3) 79 (72.3)	ADNI-2	PET: voxel volumes of FDG and florbetapir (AV-45) images	CNN	10-fold cross-validation	pMCI vs sMCI: 84.2	0.89
Donnelly-Kehoe et al. (2018) [82]	HS AD sMCI pMCI	100 (NA) 100 (NA) 100 (NA) 100 (NA)	ADNI-1	MRI: brain morphometry, demographic data, and MMSE	RF SVM AB	Train/test method: 75/25 partition	NA	0.75 0.76 0.63
Gao et al. (2018) [83]	HS AD MCI	94 (76.3) 58 (74.2) 147 (74.8)	ADNI-1	MRI and PET: hippocampal textrure features, medical history, and neuropsychological tests	GPR PLS	Train/test method: AD + HS as train set and MCI as test set + follow-up	pMCI vs sMCI: GPR:82.2 PLS:85.5	NA
Gómez-Sancho et al. (2018) [84]	HS AD sMCI pMCI	413 (NA) 326 (NA) 173 (NA) 274 (NA)	ADNI-1	MRI: regional volumetry, surface area, and cortical thickness	SVM RLR	10-fold cross-validation	pMCI vs sMCI: SVM: 61-62.5 RLR: 61.1-65	0.64-0.68 0-65-0.70
Hojjati et al. (2018) [85]	sMCI pMCI	62 (73) 18 (73.6)	ADNI-1	MRI: whole-brain cortical thickness, volumes, and connectivity matrix	SVM	9-fold cross-validation	pMCI vs sMCI: 97	0.98
Khanna et al. (2018) [86]	HS MCI	315 (NA) 609 (NA)	ADNI-1	MRI and PET: volume, clinical, and SNP measures	GBM	10 iterations of a 10-fold cross-validation	C-index (it is a generalization of the AUC ROC calculation for binary classification): 0.86	
Lin et al. (2018) [87]	HS AD sMCI pMCI	229 (NA) 188 (NA) 139 (NA) 169 (NA)	ADNI-1	MRI: hippocampal measures and whole-brain cortical volume, surface area, and cortical thickness	LASSO + ELM	*Leave-one-out* cross-validation	pMCI vs sMCI: 79.9	0.86
Liu et al. (2018) [88]	HS AD sMCI pMCI	230 (77.1) 200 (76.6) 160 (76.2) 120 (75.9)	ADNI-1	MRI: whole-brain hierarchical structural network	Multiple Kernel Boost	10-fold cross-validation	pMCI vs sMCI: 72.8	0.72
Liu et al. (2018) [89]	HS AD MCI	126 (76) 186 (75.4) 395 (74.9)	ADNI-1	MRI and PET: volume, intensity, and CSF (Aβ_42,_ t-tau y p-tau) measures	Multi-hyper graph Learning	10-fold cross-validation	pMCI vs sMCI: 74.7	0.72
Lu et al. (2018) [90]	HS AD sMCI pMCI	360 (73.4) 238 (75) 409 (74) 217 (74)	ADNI-1	MRI and PET: volume, intensity, and CSF (Aβ_42,_ t-tau y p-tau) measures	Deep NN	10-fold cross-validation	pMCI vs sMCI: 82.9	NA
Minhas et al. (2018) [91]	sMCI pMCI	54 (74.1) 65 (74.7)	ADNI-1	MRI: whole-brain volumes, surface area, cortical thickness, and neuropsychological measures	SVM	5-fold cross-validation	pMCI vs sMCI: 84.3	0.89
Popuri et al. (2018) [92]	sHS uHS pSH pMCI sMCI eDAT sDAT	360 (75.4) 52 (78.9) 18 (78.2) 205 (74.8) 431 (75) 133 (76.6) 238 (75.8)	ADNI-1	PET: glucose metabolic signal	FPDS	Independent test set	Classification of DAT+/DAT-: pMCI = 67.9 sMCI = 70.4	pMCI vs sMCI at 2, 3, and 5 years: 0.81 0.80 0.77
Sorensen et al. (2018) [93]	HS AD sMCI pMCI	100 (NA) 100 (NA) 100 (NA) 100 (NA)	ADNI-1	MRI: brain volumetry, cortical thickness, WM hipointensities, MMSE, age, and hippocampal sub-regional volumetry	Linear SVM RBF SVM	Train/test method: 60/40 partition	pMCI vs sMCI: Linear SVM: 55.6 RBF SVM: 55	NA NA
Sun et al. (2018) [94]	HS AD sMCI pMCI	162 (76.3) 137 (76) 134 (74.5) 76 (74.8)	ADNI-1	MRI: GM densities	LASSO + SVM	Train/test method: 50/50 partition	pMCI vs sMCI: 65.4	0.68
Wu et al. (2018) [95]	HS sMCI pMCI	150 (75.6) 150 (75.3) 157 (75.3)	ADNI-1	MRI: 3D brain volumes	GoogleNet CaffeNet	5-fold cross-validation	GoogleNet/CaffeNet in: pMCI: 84.7/92.3 sMCI: 67.3/72	NA
Yan et al. (2018) [96]	sMCI pMCI	50 (NA) 29 (NA)	ADNI-1	PET: 3D images	ResNet	10-fold cross-validation	pMCI vs sMCI: 82	0.81
Basaia et al. (2019) [97]	HS AD sMCI pMCI	352 (74.5) 294 (75.1) 510 (72.3) 253 (73.8)	ADNI-1	MRI: WM, GM, and CSF measures	CNN	Independent test set	pMCI vs sMCI: 74.9	NA
Cheng et al. (2019) [98]	HS AD MCI	112 (NA) 102 (NA) 192 (NA)	ADNI-1	MRI: GM volumes and CSF measures	SVM	10-fold cross-validation	pMCI vs sMCI: 76.3	0.81
Collazos-Huertas et al. (2019) [99]	sMCI pMCI	325 (75) 245 (75)	ADNI-1	MRI: volumes and cortical thickness	SVM KNN	5-fold cross-validation	pMCI vs sMCI: SVM: 76.1 KNN: 77.8	NA NA
Elahifasaee et al. (2019) [100]	HS AD sMCI pMCI	229 (76) 198 (57.5) 236 (74.9) 167 (74.9)	ADNI-1	MRI: GM density	KDA	10-fold cross-validation	pMCI vs sMCI: 65.9	0.71
Ezzati et al. (2019) [101]	HS AD sMCI pMCI	424 (74.3) 249 (74.7) 372 (72.8) 235 (73)	ADNI-1	MRI: whole-brain volumes, APOE4 genotype, and demographic measures	Ensemble Learning	10-fold cross-validation	MCI to AD at 6, 12, 24, 36, and 48 months: 63.8%, 68.9%, 74.9%, 75.3%, and 77%, respectively	NA
Gupta et al. (2019) [102]	HS AD sMCI pMCI	38 (76.7) 38 (77.1) 36 (74.2) 46 (76.7)	ADNI-1	MRI and PET: whole-brain volume, intensity and CSF (Aβ_42,_ t-tau y p-tau) measures	Mk-SVM	10-fold cross-validation	pMCI vs sMCI: 94.9	0.94
Lee et al. (2019) [103]	HS AD sMCI pMCI	415 (NA) 338 (NA) 558 (NA) 307 (NA)	ADNI-1	MRI: brain phenotypes, demographic and neuropsychological data, APOE4 genotype, and CSF measures	rDNN	5-fold cross-validation	pMCI vs sMCI at 6, 12, 18 and 24 months: 81, 81, 79 and 80 respectively	NA
Lee et al. (2019) [104]	HS AD sMCI pMCI	229 (76) 198 (75.4) 214 (75) 160 (74.9)	ADNI-1	MRI: GM volumes	rDNN + SVM	10-fold cross-validation	pMCI vs sMCI: 88.5	0.96
Lei et al. (2019) [105]	HS AD sMCI pMCI	152 (NA) 91 (NA) 98 (NA) 104 (NA)	ADNI-1	MRI: GM volumes and neuropsychological measures	SVR	10-fold cross-validation	pMCI vs sMCI: 78	NA
Li et al. (2019) [106]	NA	803 (NA)	ADNI-1/2/GO and AIBL	MRI: hippocampal volumes, cognitive, demographic and neuropsychological measures	NN	Train in ADNI-1 and test in ADNI-2&GO	MCI to AD prediction (C-index): 0.86	
Li et al. (2019) [107]	HS AD sMCI pMCI	165 (76.4) 142 (76.1) 95 (74.9) 126 (73.4)	ADNI-1	MRI: cortical thickness and volumes	SVM with RBF kernel	10-fold cross-validation	pMCI vs sMCI: 69.8	0.70
Oh et al. (2019) [108]	HS AD sMCI pMCI	230 (76) 198 (75.6) 101 (74.1) 166 (74.8)	ADNI-1	MRI data	CNN	5-fold cross-validation	pMCI vs sMCI: 73.9	NA
Pan et al. (2019) [109]	HS AD sMCI pMCI	90 (76.1) 94 (75.8) 44 (77.6) 44 (75.9)	ADNI-1	PET: intensities and connectivity measures	SVM	10-fold cross-validation	pMCI vs sMCI: 72.3	0.72
Pusil et al. (2019) [110]	sMCI pMCI	27 (71.2) 27 (74.8)	Sample collected for the study	MEG: brain connectivity matrix	MCFS + SVM with RBF kernel	Train/test method: 80/20 partition	pMCI vs sMCI: 100	NA
Spasov et al. (2019) [111]	HS AD sMCI pMCI	184 (74.6) 192 (75.6) 228 (72.2) 181 (73.7)	ADNI-1	MRI: brain volumes, demographic, neuropsychological, and genetic (APOE4) measures	CNN	Train/test method: 90/10 partition	pMCI vs sMCI: 86	0.93
Wang et al. (2019) [112]	HS AD MCI	71 (72.5) 48 (75) 60 (72.6)	ADNI-2	MRI: morphometry and WM structural connectivity	LR	10-fold cross-validation	pMCI vs sMCI: 59	0.65
Wee et al. (2019) [113]	HS AD MCI eMCI lMCI	ADNI-1/ADNI-2: 242/300 (76.9/75.6) 355/261 (76.3/75.3) 415/NA (75.9) NA/314 (72.9) NA/208 (73.7)		MRI: cortical thickness	Graph NN	10-fold cross-validation	Conversion from: eMCI to AD: 79.2 lMCI to AD: 65.2	NA
Xu et al. (2019) [114]	HS AD sMCI pMCI	165 (76.4) 142 (76.1) 95 (74.9) 126 (73.4)	ADNI-1	MRI: cortical thickness	SVM with RBF kernel	10-fold cross-validation	pMCI vs sMCI: 63.7	0.67
Zhou et al. (2019) [115]	HS AD sMCI pMCI	204 (76.1) 171 (75.5) 205 (75.1) 157 (74.8)	ADNI-1	MRI and PET: GM volumes, average intensities and SNP measures	Multi-modal Classifier	10-fold cross-validation	pMCI vs sMCI: 74..3	0.75
Zhu et al. (2019) [116]	HS AD MCI	101 (75.8) 93 (75.4) 202 (75.1)	ADNI-1	MRI and PET: GM volumes and average intensities	SVM	10-fold cross-validation	pMCI vs sMCI: 72.6	0.73
Abrol et al. (2020) [117]	HS AD sMCI pMCI	237 (74.3) 157 (75.1) 245 (72.1) 189 (74.2)	ADNI-1 ADNI-2 ADNI-3 ADNI-GO	MRI: 3D brain volumes	ResNet	Train/test method: 80/20 partition	pMCI vs sMCI: 75.1	0.78
Gao et al. (2020) [118]	HS sMCI pMCI	847 (56.9) 129 (74.8) 168 (74.8)	ADNI-1	MRI: 3D brain volumes	Age prediction + AD-NET	5-fold cross-validation	pMCI vs sMCI; 76	0.81
Giorgio et al. (2020) [119]	HS MCI	317 (NA) 272 (NA)	ADNI-1	MRI and PET: GM density, genetic, and cognitive measures	GMLVQ	10-fold cross-validation	pMCI vs sMCI: 81.4	NA
Khatri et al. (2020) [120]	HS AD MCI	57 (75.6) 53 (74.4) 77 (74.1)	ADNI-1	MRI: cortical thickness, surface area, GM volumes, MMSE, APOE4 data, and levels of Aβ42, T-tau and P-tau in CSF	SVM with RBFk Linear SVM ELM	10-fold cross-validation	pMCI vs sMCI: SVM-RBFk: 71.3 Linear SVM: 75.7 ELM: 83.4	NA NA 0.85
Lin et al. (2020) [121]	HS AD sMCI pMCI	200 (73.9) 102 (75.7) 205 (71.8) 110 (73.9)	ADNI-1	MRI and PET: volume, cortical thickness, intensity measures, APOE4 presence, and levels of Aβ42, T-tau, and P-tau in CSF	LASSO + ELM with Gaussian kernel	10-fold cross-validation	pMCI vs sMCI: 84.7	0.89
Lin et al. (2020) [122]	sMCI pMCI	124 (70.8) 40 (71.6)	ADNI-1	MRI: GM densities	SVM	4-fold cross-validation	pMCI vs sMCI: 97.3	0.98
Pan et al. (2020) [123]	HS AD sMCI pMCI	262 (NA) 237 (NA) 173 (NA) 115 (NA)	ADNI-1	MRI: 3D brain volumes	CNN + EL	5-fold cross-validation on independent test set	pMCI vs sMCI: 62	0.59
Ramon-Julvez et al. (2020) [124]	HS AD sMCI pMCI	181 (NA) 191 (NA) 227 (NA) 179 (NA)	ADNI-1	MRI data and Jacobian determinant of diffeomorphic transformations	CNN	10-fold cross-validation	pMCI vs sMCI: 89	0.94
Xiao et al. (2020) [125]	HS AD sMCI pMCI	50 (77.8) 51 (75.8) 45 (71.9) 51 (72.5)	ADNI-1	MRI: GM volumes	LR	10-fold cross-validation	pMCI vs sMCI: 72.9	NA
Xu et al. (2020) [126]	HS MCI	53 (69.6) 76 (73.7)	Sample collected for the study	MEG: brain connectivity matrix	MG2G Embedding model	Train/validation/test method: 85/10/5 partition	HS vs pMCI vs sMCI: 82 pMCI vs sMCI: 87	0.75-0.96
Yang et al. (2020) [127]	sMCI pMCI	280 (72) 70 (71.7)	ADNI-1	PET: GM densities	CNN + SVM	Train/test method: unknown partition	pMCI vs sMCI: 78.6	NA
Yee et al. (2020) [128]	sHS uHS pSH pMCI sMCI eDAT sDAT	359 (75.4) 51 (79) 19 (78.1) 210 (75) 427 (75) 135 (76.6) 237 (75.7)	ADNI-1	PET: intensity measures	CNN	5-fold cross-validation	pMCI vs sMCI: 74.7	0.81
Zhou et al. (2020) [129]	HS AD sMCI pMCI	226 (75.8) 186 (75.3) 205 (75.1) 157 (74.7)	ADNI-1	MRI and PET: GM volumes and intensity measures	SVM	10-fold cross-validation	pMCI vs sMCI: 74-76	0.74-0.76
Bae et al. (2021) [130]	HS AD sMCI pMCI	2084 (76.5) 1406 (76.2) 222 (72.2) 228 (74.2)	ADNI-1	MRI: 3D brain volume and neuropsychological measures	CNN	Train/validation/test method: 70/15/15 partition	pMCI vs sMCI: 82.4	NA
Mofrad et al. (2021) [131]	sMCI pMCI	333 (NA) 134 (NA)	ADNI-1	MRI: hippocampal entorhinal cortex, ventricles, and neuropsychological measures	Ensemble Learning	15-fold cross-validation	pMCI vs sMCI: 77	NA
Mofrad et al. (2021) [132]	sMCI pMCI	279 (NA) 279 (NA)	ADNI-1 and AIBL	MRI: hippocampal and ventricle measures	Ensemble Learning	15-fold cross-validation	pMCI vs sMCI: 78	NA
Pan et al. (2021) [133]	HS AD sMCI pMCI	242 (73.6) 237 (75) 360 (71.7) 166 (73.9)	ADNI-1	PET: 3D images	CNN	5-fold cross-validation repeated 2 times	pMCI vs sMCI: 83	0.87
Shen et al. (2021) [134]	HS AD sMCI pMCI	150 (NA) 143 (NA) 89 (NA) 86 (NA)	ADNI-1	MRI and PET: Volume, cortical thickness, intensity measures, APOE4 presence and levels of Aβ42, T-tau, and P-tau in CSF	SVM	10-fold cross-validation	pMCI vs sMCI: 75-78	0.76-0.80
Syaifullah et al. (2021) [135]	HS AD MCI	543 (NA) 359 (NA) 544 (NA)	NA-ADNI	MRI and PET data and MMSE	SVM	Train/test method: 50/50 partition	pMCI vs sMCI: 87.9	NA
Wen et al. (2021) [136]	HS AD MCI sMCI pMCI	46 (72.7) 46 (74.4) 97 (72.9) 54 (72.6) 24 (74.2)	ADNI-1	MRI: GM density	SVM	10-fold cross-validation	pMCI vs sMCI: 80	NA
Zhang et al. (2021) [137]	HS AD sMCI pMCI	275 (76.2) 280 (76.1) 251 (77.6) 162 (75.1)	ADNI-1	MRI: 3D brain volumes	CNN	Train/validation/test method: 70/15/15 partition	pMCI vs sMCI: 78.8	0.87
Zhu et al. (2021) [138]	HS AD sMCI pMCI	275 (76.2) 280 (76.1) 251 (77.6) 162 (75.1)	ADNI-1	MRI: GM, WM, and CSF measures, demographic data and APOE genotype	Temporally structured-SVM	10-fold cross-validation	pMCI vs sMCI: 85.4	0.86

*Note*. *AB* Ada-Boost, *AD* Alzheimer’s disease, *AD-NET* Age-adjust neural network, *AIBL* Australian Imaging, Biomarkers and Lifestyle Flagship Study of Aging, *ANN* Artificial neural network, AUC Area under the curve, *CNN* Convolutional neural network, *DAT* Dementia Alzheimer type, *DBM* Deep Boltzmann Machine, *DW-S2MTL* Deep-weighted subclass-based sparse multi-task learning, *EL* Ensemble learning, *eDAT* Early DAT, *ELM* Extreme learning machine, *eMCI* Early MCI, *EN* Elastic nets, *F-FDG* Fluorine 18 fluorodesoxyglucose, *FPDS* FDG-PET, *GM* Gray matter, *GMB* Gradient boosting model, *GPR* Gaussian process regression, *HS* Healthy subjects, *HLR* Hierarchical logistic regression, *ICA* Independent component analysis, *KDA* Kernel discriminant analysis, *lMCI* Late MCI, *LR* Logistic regression, *M2TL* Multimodal manifold-regularized transfer learning, *M3TL* Multi-modal multi-task learning, *MCI* Mild cognitive impairment, *MCFS* Multi-cluster feature selection, *MG2G* Multiple Graph2Gauss, *MKL* Multiple kernel learning, *MMSE* Mini Mental State Examination, *MTSRC* Multi-task sparse representation classifier, *NA* Not applicable, *nl-SVM-RBFk* Non-linear SVM with radial basis function kernel, *NN* Neural network, *OPLS* Orthogonal partial least squares, *PBL-McqRBFN* Projection-based learning for meta-cognitive q-Gaussian radial basis function network, *PLS* Partial least squares, *pMCI* Progressive MCI, *rDNN* Randomized deep neural network, *Res-Net* deep residual neural network, *RF* Random forest, *RLR* Regularized logistic regression, *RUSRF* Random under sampled random forest, *sDAT* Stable DAT, *SM2TLC* Sparse multimodal manifold-regularized transfer learning classification, *sMCI* Stable MCI, *SNN* Spiking neural network, *SNP* Single-nucleotide polymorphisms, *ss* Sample selection, *SVM* Support vector machine, *VFI* Voting feature intervals, *WM* White matter, *wmSRC* Weighted multi-modality sparse representation-based classification

MRI was the most common kind of neuroimaging used (in 76 out of 116 studies), followed by PET (11 studies), 26 studies included data from both techniques (MRI and PET), two studies used magnetoencephalography (MEG) data, and one study used MRI and MEG data.

Regarding the source of the datasets, 107 out of 116 studies used the ADNI database in any of its versions (ADNI-1, 2, 3, or GO) to obtain samples of healthy, MCI, and AD subjects. Of the remaining eight studies, three used data from AddNeuroMed (https://consortiapedia.fastercures.org/consortia/anm/) database, one used the Australian Imaging, Biomarker & Lifestyle Flagship Study of Ageing (AIBL) database, and four collected their own data. Li et al. [108] used both ADNI and AIBL.

Although almost all studies used the same database, the cohorts selected varied across them. Most articles (66 out of 116 studies) divided their participants into four groups: healthy controls, stable MCI patients (sMCI), progressive MCI patients (pMCI), and AD patients. 21 articles selected three cohorts of MCI, AD, and healthy subjects, although in order to predict the progression to AD dementia, they had to distinguish between pMCI and sMCI patients. The remaining 29 studies used different groups of participants: 21 only sMCI and pMCI, six had healthy controls, and MCI with two of them separating sMCI and pMCI. Wee et al. [113] differentiated between early and late MCI, and Li et al. [106] did not specify the cohorts selected.

The sample size also varied across studies. Wee et al. [37] has the smallest sample with 27 subjects, and Bae et al. [130] has the largest sample with 3940 subjects; the mean sample size was 546 participants. The sample size follows an ascendant trend across years, which may be attributed to the increased data availability in the ADNI database. The mean age ranged from 56 to 79 years old. Although 30 studies did not include the mean age of the sample, they used an ADNI database, and therefore, the age range might be similar to the rest of the studies. The variations in age between studies may be due to differences in participant selection and the moment when the study was conducted (since the ADNI database has been incorporating more data over the years).

As for feature selection, the most common were whole-brain volumes, selected in 70 articles, and intensity measurements of glucose metabolism, selected in 31 PET studies, also 16 studies included genetic features (APOE4 genotype). Other selected features were neuropsychological test results (18 out of 116 studies) and demographic variables such as age (15 out of 116 studies). 42 studies only used one type of features such as 3D MRI data or whole-brain gray matter volumes, and 74 studies selected two or more different types of features. As for the algorithm results, the most useful areas to discriminate between AD patients and healthy subjects or sMCI, were mainly located in the temporal, parietal, and frontal lobes. In particular, the most relevant regions were the hippocampus, amygdala, entorhinal cortex, precuneus, cingulate gyrus, and rostral and caudal areas of the medial frontal lobe.

Regarding the ML methods used to classify the patients and detect probable MCI to AD dementia progression, the most popular were those based on support vector machine (SVM). SVM was used in 60 out of the 116 studies; this method is a supervised ML algorithm that has demonstrated its utility in neuroimaging-based applications, especially in the classification of future clinical outcomes [139]. SVM takes every measurement from every subject as a single point in a multidimensional space, with the number of dimensions being the total number of features of that particular dataset (for example, 93 gray matter volumes from regions of interest). The algorithm then finds the maximal margin separating hyperplane that optimally differentiates groups of data points representing different classes (e.g., pMCI vs. sMCI, or AD vs. HC). The data instances closest to the group boundaries are the support vectors and are, by definition, the ones that determine the position of the hyperplane. The mapping into a higher dimensional space is done by a kernel function, usually polynomial or Gaussian [26]. The SVM algorithm is trained with labeled data (indicating whether the data belongs to a healthy person, sMCI, pMCI, or AD dementia patient, for example) to generate this multidimensional space. Once the model has been trained, we can introduce a new subject with MCI and it will be classified in the multidimensional space into the boundaries of one of the previously defined groups (i.e., sMCI, AD dementia). For example, if the new MCI patient is classified as belonging to the AD or pMCI group, we can infer that this subject is more likely to develop a future AD dementia due to being more similar to subjects in that group. The different groups for classification will depend on the specific methodology of each study.

The combination of SVM with other methods allows to improve feature selection and to avoid overfitting of data, and this will facilitate the generalization of the model (i.e., achieving high accuracy when applied to different datasets). For example, Thung et al. [71] used SVM with multiple kernels (linear, Gaussian, and polynomial) after feature selection with least squares and logistic elastic net regressions and also matrix completion with label-guided low-rank matrix completion method. On the other hand, Toussaint et al. [36] used non-linear SVM with Gaussian Radial Basis Function kernel but only after a two-sample *t* test and a spatial independent component analysis, performed for the detection of glucose metabolism and characteristic region patterns of AD patients. Other classification methods used were random forest or neural networks that can have different architectures, but the most commonly used for image classification tasks where convolutional neural networks, applied in 16 studies.

As for validation methods, cross-validation was selected in 72 studies, with different numbers of folds and/or iterations. Cross-validation consists in dividing the sample into two sets, one to train the algorithm (training set) and another one for validation (testing set). This partition can be done several times by changing the train/test split of the data, and the accuracy of each iteration can be averaged to obtain a more robust quantification of the model performance instead of just validating the model on one test sample. Another validation method is the leave-one-out cross-validation, selected in 13 studies. In this case, the model is trained with all the data except for one data point, then it tries to classify the data point left out and does the same with the rest of the sample in subsequent iterations. The train/test method was selected in 27 studies with different percentages of data partitions. Finally, three studies validated the model on an independent test set and Ardekani et al. [64] used “out of bag” as a validation method.

The results of ML classification algorithms can be assessed based on their sensitivity (percentage of correctly detected pMCI patients or true positive ratio) and specificity (percentage of healthy or sMCI subjects correctly identified or true negative ratio), or accuracy (percentage of correctly classified subjects). By changing the decision threshold of the classifier, we can compensate the ratio between true positive/true negative and generate a graphic representation of that ratio, or what is known as the receiver operating characteristic (ROC) curve [140]. The calculation of the area under the ROC curve (AUC ROC) represents a good quantitative index to compare the classification models, since it indicates the ability of the model to predict both the presence and non-presence of disease, or in this case, the progression or lack of progression from MCI to AD dementia [141]. An AUC ROC of one implies a perfect classification of every subject in the sample. The maximum accuracies achieved by every study in the prediction of AD dementia progression from MCI patients or the accuracy of the method in discriminating between a progressive/stable MCI are shown in the “Results” column of Table 3; the AUC coefficient is presented when available.

The mean accuracy of studies that used MRI as neuroimaging technique was 74.5% and 76.9% for studies that selected PET scans. The combination of both techniques achieved even better results with a mean accuracy of 77.5%. AUC ROC results follow a similar pattern with a mean AUC of 0.79, 0.80, and 0.80 for MRI, PET, and MRI+PET, respectively. López et al. [69] used MRI and MEG and achieved an accuracy of 100 and an AUC of 0.97 but with a sample of 33 subjects and finally Pusil et al. [110] (with 54 subjects) and Xu et al. [126] (with 129 subjects) used MEG with accuracies of 100% and 87%, respectively.

Considering the two most popular classification methods (SVM and CNN), the mean accuracies were 75.4% for SVM and 78.5% for CNN. The best results with the SVM algorithm were obtained again by Pusil et al. [110] with 100% accuracy, but using a small sample of 54 that makes the model hardly generalizable. In studies with bigger samples, Guerrero et al. [49] had the highest accuracy results (97.2% with 511 subjects) followed by Lin et al. [122] (97.3% with 164 subjects). Finally, although 43 studies did not report AUC ROC values, the highest reported was 0.98 in Lin et al. [122] followed by 0.98 in Hojjati et al. [85] with 80 subjects. The lowest accuracy reported was found in the study by Plant et al. [24] (50%), who included a small sample size [63] and is the oldest study of all the analyzed, making the comparison between studies difficult. The second-lowest accuracy was achieved by Sorensen et al. [93] (55% with 400 subjects); in this case, the study was part of a data science competition (Kaggle) where data was already preprocessed for all participants and could not be manipulated by the authors. The lowest AUC ROC reported was found in Liu et al. [45] (0.53 and 414 subjects) using SVM and in Pan et al. [123] (0.59 with 787 subjects) with CNN.

Finally, the interpretability analysis is shown in Table 4. Most of the studies presented results of *specificity and sensitivity* (113 out of 116), about half of them (60 out of 116) made an *analysis of confounds*, but only Lebedev et al. [50], Li et al. [106], and Syaifullah et al. [135] complied with the *generalizability* sublevel, testing their model in different datasets. All of the studies performed a *stability* measurement of their model, and only 25 did not specify which features were the most *important* for the classification task. On the other hand, less than half of the studies (51 out of 116) presented their results along with some kind of *visualization* of the most relevant brain areas for the prediction of MCI conversion. Finally, the comparison of the results with the existing *literature* was done in all of the studies except for four of them.

**Table 4:** Analysis of the interpretability based on Kohoutová et al. [23]

Author (year)	Model			Feature			Biology
	SEN/SPE	GN	AC	ST	IMP	VIS	LIT
Plant et al. (2010) [24]	✓	-	✓	✓	✓	-	✓
Chincarini et al. (2011) [25]	✓	-	✓	✓	✓	-	✓
Costafreda et al. (2011) [26]	✓	-	✓	✓	✓	✓	✓
Filipovych et al. (2011) [27]	✓	-	-	✓	✓	✓	✓
Hinrichs et al. (2011) [8]	✓	-	-	✓	✓	✓	✓
Westman et al. (2011) [28]	✓	-	-	✓	✓	✓	✓
Wolz et al. (2011) [29]	✓	-	-	✓	✓	✓	✓
Zhang et al. (2011) [30]	-	-	-	✓	✓	✓	✓
Batmanghelich et al. (2012) [31]	✓	-	-	✓	✓	-	✓
Cheng et al. (2012) [32]	✓	-	-	✓	-	-	✓
Cho et al. (2012) [33]	✓	-	-	✓	✓	✓	✓
Gray et al. (2012) [34]	✓	-	✓	✓	✓	✓	✓
Li et al. (2012) [35]	✓	-	-	✓	✓	✓	✓
Toussaint et al. (2012) [36]	✓	-	✓	✓	✓	✓	✓
Wee et al. (2012) [37]	✓	-	✓	✓	✓	✓	✓
Ye et al. (2012) [38]	✓	-	-	✓	✓	-	✓
Zhang et al. (2012) [9]	✓	-	-	✓	✓	✓	✓
Adaszewski et al. (2013) [39]	✓	-	-	✓	✓	✓	✓
Aguilar et al. (2013) [40]	✓	-	-	✓	✓	-	✓
Babu et al. (2013) [41]	✓	-	-	✓	✓	-	✓
Casanova et al. (2013) [42]	✓	-	-	✓	✓	-	✓
Cheng et al. (2013) [43]	✓	-	-	✓	-	-	✓
Liu, M. et al. (2013) [44]	✓	-	-	✓	-	-	✓
Liu, X. et al. (2013) [45]	✓	-	-	✓	✓	✓	✓
Wee et al. (2013) [46]	✓	-	-	✓	✓	-	✓
Young et al. (2013) [47]	✓	-	-	✓	-	-	-
Apostolova et al. (2014) [48]	✓	-	✓	✓	✓	-	✓
Guerrero et al. (2014) [49]	✓	-	✓	✓	✓	-	✓
Lebedev et al. (2014) [50]	✓	✓	-	✓	✓	✓	✓
Liu, M. et al. (2014) [51]	✓	-	-	✓	✓	✓	✓
Liu, F. et al. (2014) [52]	✓	-	-	✓	-	-	-
Min et al. (2014) [53]	✓	-	✓	✓	-	-	-
Suk et al. (2014) [54]	✓	-	✓	✓	✓	-	✓
Tong et al. (2014) [55]	✓	-	✓	✓	✓	✓	✓
Cabral et al. (2015) [56]	✓	-	-	✓	✓	✓	✓
Cheng et al. (2015) [57]	✓	-	✓	✓	-	-	-
Cheng et al. (2015) [58]	✓	-	✓	✓	✓	✓	✓
Moradi et al. (2015) [59]	✓	-	-	✓	✓	-	✓
Raamana et al. (2015) [60]	✓	-	✓	✓	-	✓	✓
Ritter et al. (2015) [61]	✓	-	-	✓	✓	-	✓
Salvatore et al. (2015) [62]	✓	-	-	✓	✓	✓	✓
Xu et al. (2015) [63]	✓	-	✓	✓	-	-	✓
Ardekani et al. (2016) [64]	✓	-	✓	✓	✓	-	✓
Collij et al. (2016) [66]	✓	-	✓	✓	✓	✓	✓
Li et al. (2016) [67]	✓	-	✓	✓	✓	✓	✓
Liu et al. (2016) [68]	✓	-	✓	✓	-	-	✓
López et al. (2016) [69]	✓	-	-	✓	✓	-	✓
Suk et al. (2016) [70]	✓	-	✓	✓	✓	✓	✓
Thung et al. (2016) [71]	✓	-	✓	✓	✓	-	✓
Vasta et al. (2016) [72]	✓	-	✓	✓	✓	-	✓
Zhang et al. (2016) [73]	✓	-	-	✓	✓	-	✓
Zhang et al. (2016) [74]	✓	-	-	✓	✓	-	✓
Ҫitak-Er et al. (2017) [75]	✓	-	-	✓	✓	✓	✓
Hojjati et al. (2017) [76]	✓	-	✓	✓	✓	✓	✓
Long et al. (2017) [77]	✓	-	-	✓	✓	-	✓
Mathotaarachchi et al. (2017) [78]	✓	-	✓	✓	✓	✓	✓
Suk et al. (2017) [79]	✓	-	✓	✓	✓	✓	✓
Tong et al. (2017) [80]	✓	-	-	✓	✓	✓	✓
Choi et al. (2018) [81]	✓	-	✓	✓	✓	-	✓
Donnelly-Kehoe et al. (2018) [82]	✓	-	✓	✓	✓	-	✓
Gao et al. (2018) [83]	✓	-	-	✓	✓	-	✓
Gómez-Sancho et al. (2018) [84]	✓	-	-	✓	✓	-	✓
Hojjati et al. (2018) [85]	✓	-	-	✓	✓	✓	✓
Khanna et al. (2018) [86]	-	-	✓	✓	✓	-	✓
Lin et al. (2018) [87]	✓	-	-	✓	✓	✓	✓
Liu et al. (2018) [88]	✓	-	-	✓	✓	✓	✓
Liu et al. (2018) [89]	✓	-	✓	✓	-	-	✓
Lu et al. (2018) [90]	✓	-	-	✓	-	-	✓
Minhas et al. (2018) [91]	✓	-	-	✓	-	-	✓
Popuri et al. (2018) [92]	✓	-	✓	✓	✓	-	✓
Sorensen et al. (2018) [93]	✓	-	-	✓	-	-	✓
Sun et al. (2018) [94]	✓	-	✓	✓	✓	✓	✓
Wu et al. (2018) [95]	✓	-	✓	✓	-	-	✓
Yan et al. (2018) [96]	✓	-	-	✓	-	-	✓
Basaia et al. (2019) [97]	✓	-	✓	✓	-	-	✓
Cheng et al. (2019) [98]	✓	-	✓	✓	✓	-	✓
Collazos-Huertas et al. (2019) [99]	✓	-	✓	✓	✓	-	✓
Elahifasaee et al. (2019) [100]	✓	-	✓	✓	✓	✓	✓
Ezzati et al. (2019) [101]	✓	-	✓	✓	-	-	✓
Gupta et al. (2019) [102]	✓	-	-	✓	✓	-	✓
Lee et al. (2019) [103]	✓	-	✓	✓	✓	-	✓
Lee et al. (2019) [104]	✓	-	-	✓	✓	✓	✓
Lei et al. (2019) [105]	✓	-	-	✓	✓	✓	✓
Li et al. (2019) [106]	✓	✓	✓	✓	✓	✓	✓
Li et al. (2019) [107]	✓	-	✓	✓	-	-	✓
Oh et al. (2019) [108]	✓	-	✓	✓	✓	✓	✓
Pan et al. (2019) [109]	✓	-	-	✓	-	-	✓
Pusil et al. (2019) [110]	✓	-	-	✓	✓	-	✓
Spasov et al. (2019) [111]	✓	-	-	✓	✓	-	✓
Wang et al. (2019) [112]	✓	-	✓	✓	-	-	✓
Wee et al. (2019) [113]	✓	-	✓	✓	✓	✓	✓
Xu et al. (2019) [114]	✓	-	✓	✓	✓	✓	✓
Zhou et al. (2019) [115]	✓	-	✓	✓	✓	✓	✓
Zhu et al. (2019) [116]	✓	-	✓	✓	✓	✓	✓
Abrol et al. (2020) [117]	✓	-	✓	✓	✓	✓	✓
Gao et al. (2020) [118]	✓	-	-	✓	-	-	✓
Giorgio et al. (2020) [119]	✓	-	-	✓	✓	-	✓
Khatri et al. (2020) [120]	✓	-	✓	✓	✓	✓	✓
Lin et al. (2020) [121]	✓	-	-	✓	✓	-	✓
Lin et al. (2020) [122]	✓	-	✓	✓	✓	✓	✓
Pan et al. (2020) [123]	-	-	-	✓	✓	-	✓
Ramon-Julvez et al. (2020) [124]	✓	-	-	✓	-	-	✓
Xiao et al. (2020) [125]	✓	-	-	✓	-	-	✓
Xu et al. (2020) [126]	✓	-	-	✓	✓	✓	✓
Yang et al. (2020) [127]	✓	-	✓	✓	-	-	✓
Yee et al. (2020) [128]	✓	-	✓	✓	✓	✓	✓
Zhou et al. (2020) [129]	✓	-	✓	✓	✓	✓	✓
Bae et al. (2021) [130]	✓	-	-	✓	✓	✓	✓
Mofrad et al. (2021) [131]	✓	-	✓	✓	✓	-	✓
Mofrad et al. (2021) [132]	✓	-	✓	✓	✓	-	✓
Pan et al. (2021) [133]	✓	-	✓	✓	✓	✓	✓
Shen et al. (2021) [134]	✓	-	✓	✓	✓	-	✓
Syaifullah et al. (2021) [135]	✓	✓	✓	✓	✓	-	✓
Wen et al. (2021) [136]	✓	-	✓	✓	✓	-	✓
Zhang et al. (2021) [137]	✓	-	✓	✓	✓	-	✓
Zhu et al. (2021) [138]	✓	-	✓	✓	✓	✓	✓
**Total (116)**	**113**	**3**	**60**	**116**	**91**	**51**	**112**

*Note.* This table shows an interpretability analysis performed for each study selected in our review following the framework proposed by Kohoutová et al. [23]. Presence (**✓)** or absence (-) of the different sublevels assessments. Behavioral analysis, representational analysis, and invasive studies sub-levels are not applicable to this type of study by its definition. *SEN* Sensitivity, *SPE* Specificity, *GN* Generalizability, *AC* Analysis of confounds, *ST* Stability, *IMP* Importance, *VIS* Visualization, *LIT* Literature

## Discussion

In this systematic review, we analyzed 116 studies, conducted over the last 10 years, which used neuroimaging data to predict conversion to AD dementia from MCI using ML algorithms. The complexity of neuroimaging results and the amplitude of the deterioration and symptoms present in multiple areas and functions in AD, make its detection very complex in patients with MCI by simply visualizing a single patient neuroimaging data. Nevertheless, using the publicly available data collected over the last decades, together with the newly developed ML algorithms, researchers can not only distinguish the brains of AD patients and healthy people with high accuracy, but also predict MCI patient’s disease progression (i.e., whether a MCI patient will progress to AD dementia or remain stable in the future). This information is highly valuable for clinicians in order to achieve a more accurate prognosis and therefore set treatment plans that can slow down the development of the disease and prevent higher degrees of cognitive impairment.

The 116 studies analyzed reached different levels of accuracy using classification methods based on ML algorithms. Only 24 studies focused exclusively on predicting MCI progression, most studies also tried to find the main differences between healthy controls and AD patients. The specific search for AD biomarkers is much more abundant in the literature than predicting progression from a MCI or even from healthy subjects [142]. In any case, in the studies that carried out both tasks and in studies that focused on the prediction of AD progression, the distinction between controls and AD was always more accurate than the distinction between pMCI vs. sMCI, showing the difficulty of finding biomarkers before functional impairment/dementia appear.

One of the main challenges of this review was to compare studies with highly variable methodologies including different samples, preprocessing techniques, types of neuroimaging data, and also different classification and validation methods. Still, studies that achieved higher levels of accuracy have in common the use of multimodal and multidimensional data combined with increasingly complex classification methods. Easy-to-implement algorithms, such as those based on SVM, are leaving room for more complex algorithms based on deep learning paradigms such as neural networks, capable of identifying dementia-associated subtle changes of brain morphology in a way able to increase the number of correctly classified subjects. All methods seemed to benefit from the inclusion of demographic variables and cognitive measurements, and even genetic variables if these were available. Nevertheless, in order for these techniques to be able to help clinicians in their everyday practice, a balance is needed between the most advanced data and algorithms that achieve the higher performance, and the data and methods that might be available in the clinical practice. In this sense, future studies might need to focus more on achieving high performance using large datasets with more essential (and easily obtainable) data such as structural MRI, demographic, and cognitive results.

Regarding the sample, most studies use the publicly available data from the ADNI database. This database is still incorporating new data and the most recent studies even use ADNI-2 and ADNI-3 [113, 117]. The main problem of the studies performed 10 years ago is their smaller sample size. Furthermore, even if two studies report similar accuracies, a study with a bigger sample size will have results that are more generalizable. For example, Plant et al. [24] and Popuri et al. [92] obtained similar accuracies of 75% and 79% of correct classifications, respectively, but Popuri et al. [92] used a sample of about 30 times larger. Upon review of the recent developments in the field, it is apparent that as these patient populations continue to age and their disease progression matures and is clinically diagnosed, the inclusion of their ongoing neuroimaging data will enrich these public databases, thereby enabling improved validation of classification methods. Nevertheless, it should be noted that a follow-up of 2 or 3 years might not be long enough to detect progression to AD dementia. Therefore, subjects considered as stable MCI or even as healthy subjects might, in fact, progress to AD dementia in the long term. This problem will always be present with the inclusion of new cases in the ADNI database, but the follow-ups recorded will be increasingly long-lasting, thus being more useful. On the other hand, in order for these methods to be clinically useful, the models have to be tested, not only in big samples, but also in more variable and diverse groups of people, other than the ADNI sample. This approach is highlighted by Lebedev et al. [50], for example, who applied the model to the ADNI and then to the AddNeuroMed cohort, achieving similar performance and accuracy results in both, making it more robust for future clinical implementation.

Another interesting result from the inclusion of neuroimaging data in ML algorithms is the possibility of finding out which brain regions are more relevant to predict the conversion from MCI to AD dementia. In this case, the highlighted regions as informed by the algorithms (hippocampus, amygdala, entorhinal cortex, precuneus, cingulate gyrus, and medial frontal lobe) have been widely validated by the scientific literature as relevant in the progression to AD [8, 33, 34, 143–147]. This coincidence between the literature and the algorithm results supports the notion that the classification methods can detect differences between groups based on relevant neuroimaging features.

In terms of accuracy, although the algorithms are useful and able to discriminate the brain characteristics of AD, the performance of the algorithms are far from being specific enough to leave complete diagnosis in the hands of automated methods, so the judgment of a clinical professional will remain crucial in the near future. Also, clinical criteria can achieve sometimes similar results in terms of predicting the conversion from MCI to AD dementia 1 year before its onset [148]. Nevertheless, computer-aided diagnosis, when implemented in the clinical practice, will offer a faster, easier to perform, and earlier detection way of predicting the potential progression to AD dementia in MCI patients. Therefore, the automated methods discussed above present a low-cost approach that can be useful as a first approximation, a method to discriminate ambiguous cases, and a support tool for large datasets.

Clinical research is moving towards a broader and more open context where professionals from very different disciplines might be interested in these types of studies. As such, it is important to present the results from complex neuroimaging classification studies as clearly as possible. The framework to interpret ML models provided by Kohoutová et al. [23] is a helpful starting point for this purpose. Most or all of the studies reviewed here included information about the specificity and sensitivity (model level), the stability of the models, and the most important features selected (feature level), along with a comparison with the previous literature (biology level). However, there are some important issues that should be addressed in future studies such as the inclusion of visualizations of the most relevant brain areas to predict MCI conversion, an adequate analysis of confounds, and generalization methods. These specific improvements would provide more comprehensive and comparable studies.

### Limitations

Regarding the limitations of the review, it is worth mentioning that we did not include methodological details such as the preprocessing methods to obtain the neuroimaging results or the mathematical development of the algorithms. This information could have provided a better understanding of each model's performance and how the data is classified to differentiate between groups, but these deep methodological analyses were out of the scope of this review given its more clinically oriented focus.

## Conclusions

The recent trend in research to find diagnostic automation methods presents great potential in the early detection of neurodegenerative diseases. Since structural changes appear before the clinical symptoms manifest, there is a valuable period of time in which the morphological and functional changes in the brain can be detected and, therefore, used to predict and provide clinical treatment to slow down the future development of a neurological disease.

Research in this field is still rapidly advancing, new increasingly complex algorithms continue to be developed, and access to higher levels of computational capacity is also increasing, as well as the precision and resolution of neuroimaging techniques. In the future, we can expect faster, more precise, and more efficient classification methods that may be directly incorporated into the neuroimaging techniques themselves that enable the generation of a diagnostic hypothesis with a simple scan of a patient’s brain. However, the challenge to translate this knowledge into daily practice remains. This challenge will be overcome on the one hand by increasing the generalizability of the classification methods as they are applied to more diverse samples and, on the other hand, by finding the trade-off between the higher precision achieved when including complex information and a sufficient performance using only the clinical data commonly available for the clinicians. Thus, future studies should focus on obtaining good results using data easily available in the clinical practice (structural MRI, demographic data, and cognitive results, for example) and making their models as much generalizable as possible using more diverse and inclusive samples.

## Data Availability

Not applicable.

## Abbreviations

AUC: Area under the curve
AD: Alzheimer’s disease
ADNI: Alzheimer’s disease neuroimage initiative
HC: Healthy controls
MCI: Mild cognitive impairment
ML: Machine learning
MRI: Magnetic resonance image
PET: Positron emission tomography
pMCI: Progressive mild cognitive impairment
ROC: Receiver operating characteristic
sMCI: Stable mild cognitive impairment
SVM: Support vector machine
